# Primary Varicella in an Adult Patient With Diabetes

**DOI:** 10.7759/cureus.93301

**Published:** 2025-09-26

**Authors:** Abigail Hendrie, Anh Hoang, Jared Marshall

**Affiliations:** 1 David Geffen School of Medicine, University of California Los Angeles, Los Angeles, USA; 2 Emergency Medicine, University of California Los Angeles, Los Angeles, USA

**Keywords:** adult-onset chickenpox, diabetes mellitus, herpes zoster virus, immunocompromised, primary varicella, varicella vaccine, vesicular rash

## Abstract

Although the introduction of the varicella vaccine has significantly reduced the incidence of primary varicella in adults, individuals with compromised immune systems, such as those with diabetes mellitus, remain at heightened risk for severe disease. We report the case of a 44-year-old male with a history of diabetes mellitus who presented to the emergency department with a diffuse, generalized rash associated with pruritus, burning sensation, and numbness and tingling. A polymorphic rash diffusely involving the face, trunk, extremities, scalp and palms emerged, beginning with erythematous macules and sequentially developing into papules, vesicles, and crusted lesions. A clinical diagnosis of primary varicella was made based on the characteristic rash and the patient’s immunocompromised status, which was confirmed with antibody testing. He was treated with valacyclovir, corticosteroids, multimodal pain control, and antihistamines. This case highlights the continued risk of primary varicella in adults and the importance of considering it within the differential diagnosis, particularly in patients with immune dysfunction.

## Introduction

Since the introduction of the varicella vaccine in 1995, the incidence of primary varicella (chickenpox) in adults has significantly declined. According to the Centers for Disease Control and Prevention (CDC), widespread childhood vaccination has led to a more than 90% reduction in varicella cases, hospitalizations, and deaths across all age groups [1]. However, unvaccinated individuals and those with weakened immune systems remain susceptible to severe disease. Immunocompromised patients are at an increased risk for disseminated disease and complications due to their impaired immune response [2]. An immunocompromised state generally refers to patients with underlying malignancy, HIV, or patients on high-dose steroids or other immunomodulatory medications, but may also involve those with sub-optimally controlled diabetes. Pregnant women are also a category of patients who are at risk for severe disease. While varicella is typically mild in children, adults who contract the virus are at significantly higher risk for severe complications, including varicella pneumonia, encephalitis, and secondary bacterial infections [3]. Mortality rates are also higher in adults. According to a study investigating deaths caused by varicella between 2008 and 2011, 71% were in persons aged ≥50 years [4].

Primary varicella-zoster virus (VZV) infection occurs through respiratory droplets or direct contact with vesicular fluid. Following mucosal entry, VZV undergoes primary replication in the upper respiratory tract, followed by hematogenous and lymphatic spread, leading to viral seeding in the skin [5]. The characteristic vesicular rash results from viral replication in keratinocytes, immune-mediated cytolysis, and intraepidermal vesicle formation [6]. VZV also establishes latency in dorsal root ganglia, where it may later reactivate as herpes zoster (shingles).

This case describes a middle-aged diabetic patient who developed a widespread vesicular rash consistent with primary varicella infection. His presentation was notable for a preceding viral prodrome, a polymorphic rash in various stages of healing, and associated neuropathic symptoms.

## Case presentation

A 44-year-old male with a history of diabetes mellitus presented to the emergency department reporting a one-week history of a generalized, persistent rash. Associated symptoms included pruritus, numbness and tingling, and burning sensation. Prior to the eruption of the rash, he had sore throat and chills. 

**Figure 1:**
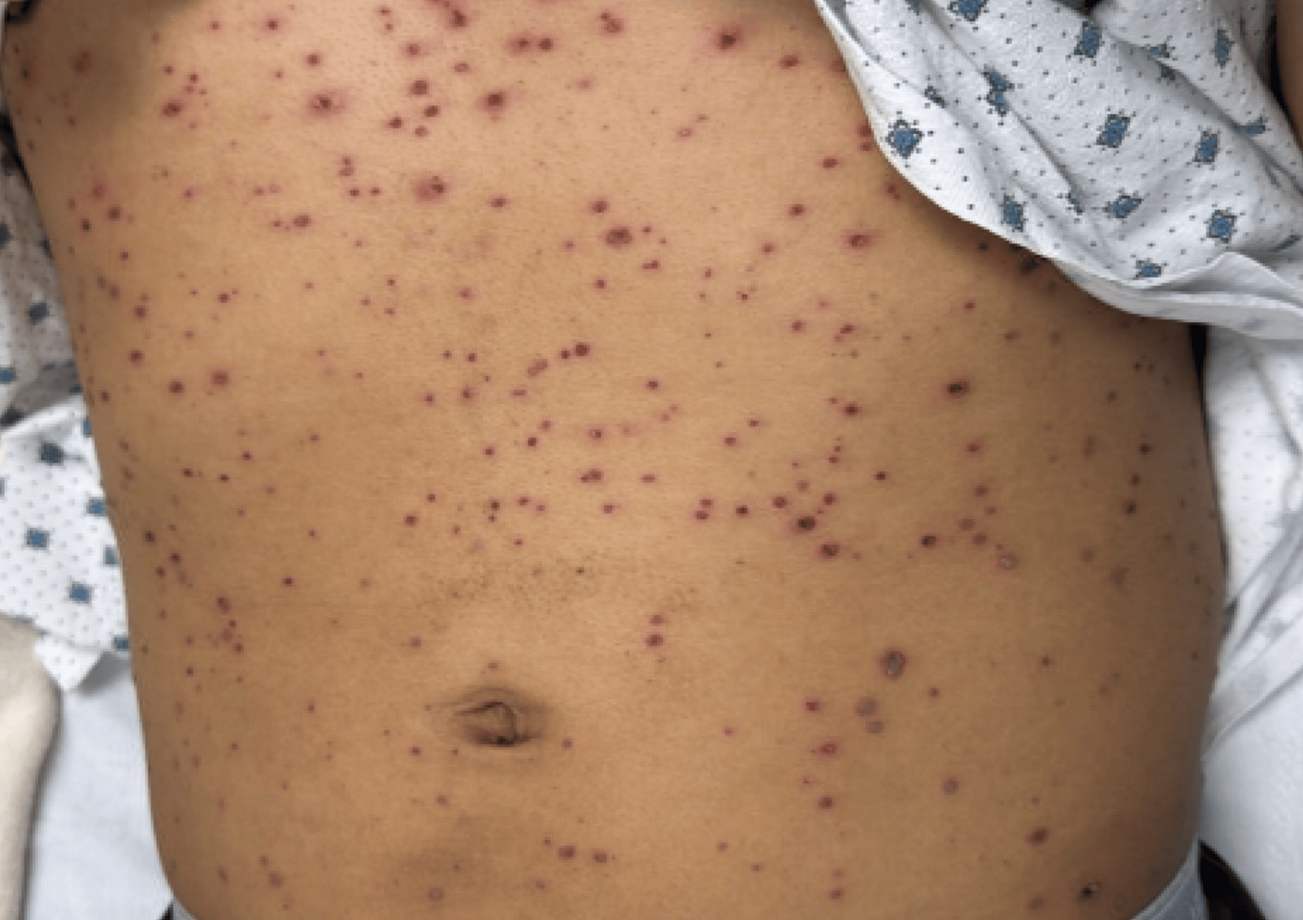
Numerous lesions in varying stages of evolution on the anterior chest and abdomen. The distribution demonstrates centripetal spread, with dense involvement of the trunk.

**Figure 2:**
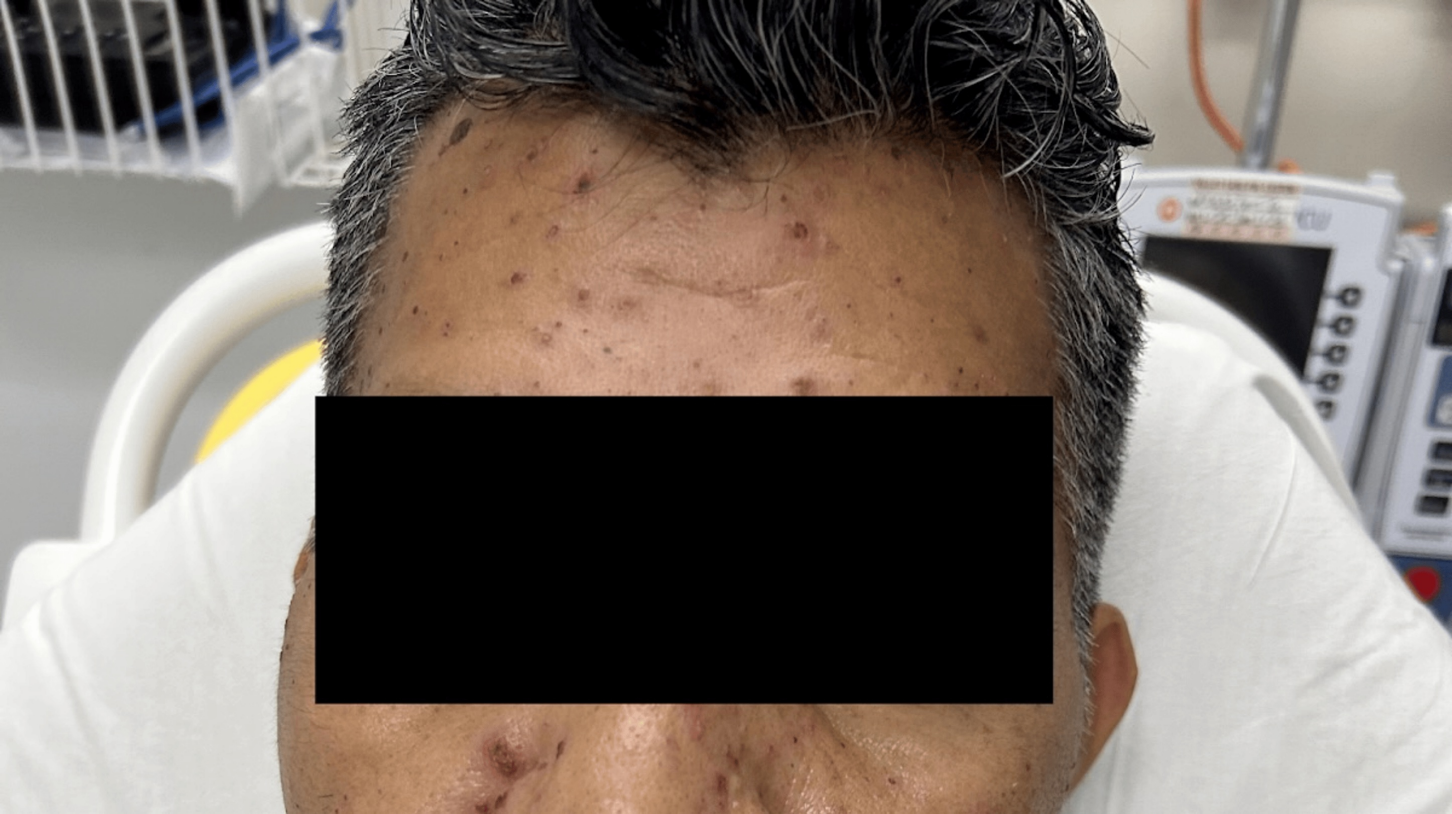
Multiple vesicles and crusted papules on the face.

**Figure 3:**
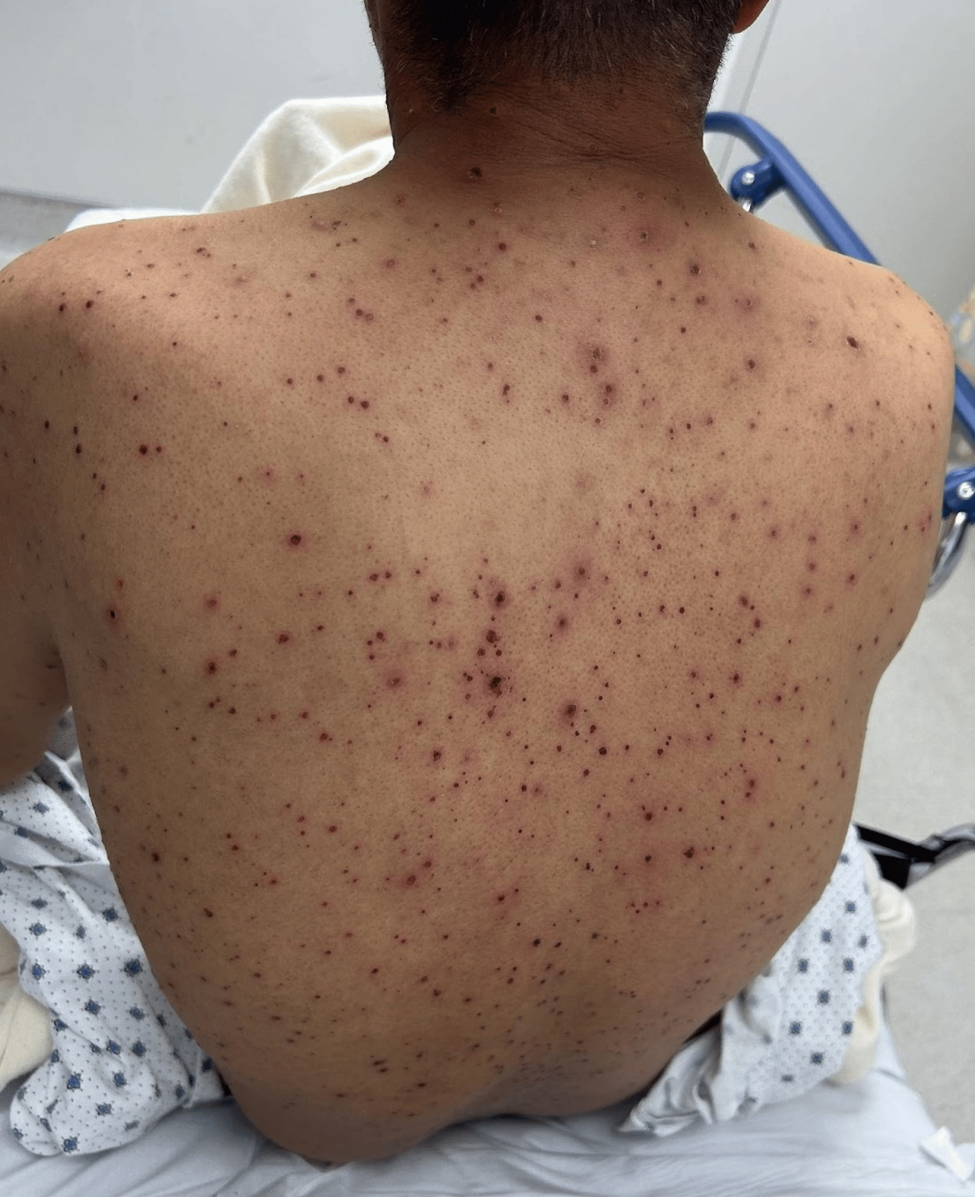
Numerous discrete erythematous papules, vesicles, and crusted lesions scattered across the upper and lower back. Truncal predominance and diffuse distribution are consistent with the classic pattern of varicella-zoster virus.

**Figure 4:**
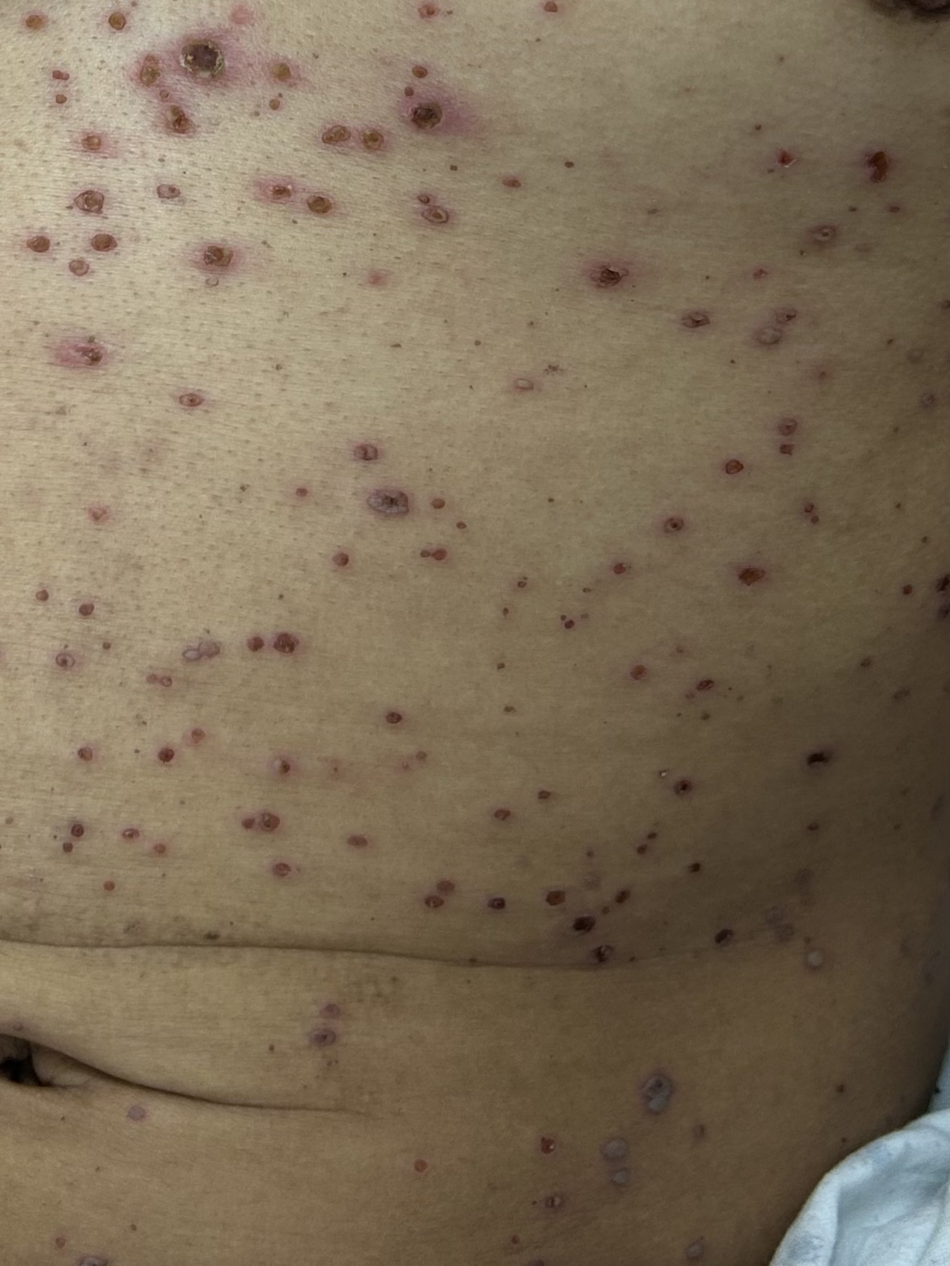
Close-up view of lesions showing multiple umbilicated vesicles, erythematous papules, and crusted erosions.

The rash initially appeared as erythematous macules on the trunk, which subsequently spread to the face, extremities, scalp, and palms. On examination, lesions in different stages of healing were noted, including papules, clear vesicles, and crusted lesions (Figures 1-4).

The polymorphic appearance of the rash, along with the preceding viral prodrome, raised suspicion for primary varicella infection. The patient was unsure of his varicella vaccination history. Laboratory testing revealed that both IgM and IgG antibodies to VZV were positive, a serologic pattern consistent with acute or recent primary infection (Table 1). IgM positivity typically emerges during the first few days of rash onset, while IgG seroconversion may begin shortly after and persists lifelong, supporting the diagnosis of a recent primary varicella infection rather than reactivation.

**Table 1:** VZV IgG and IgM serologic testing results. VZV: varicella-zoster virus

Test	Result	Reference ranges	Interpretation
VZV Ab IgG	5.17	Negative: < 1.00; Positive: ≥ 1.00	Positive
VZV Ab IgM	2.33, 2.19	Negative: 0.00 - 0.90; Equivocal: 0.91 - 1.09; Positive: ≥ 1.10	Positive

The patient was treated with valacyclovir and a brief course of steroids, with attention paid to his diabetes and caution exercised to avoid poor glycemic control. While corticosteroids are not routinely indicated in uncomplicated varicella, their selective use in adults may be considered to mitigate inflammation, reduce symptom burden, and potentially shorten the duration of cutaneous disease. We recognize that adjunctive use of corticosteroids in VZV infection is not routinely recommended and remains a highly individualized decision. While high-quality trials in disseminated VZV are lacking, corticosteroids added to antivirals for herpes zoster have demonstrated a modest reduction in pain and quality-of-life metrics in the acute setting, which is relevant to our practice in Emergency Medicine [7-9]. Furthermore, there was low concern for superimposed bacterial infection or pulmonary involvement, and steroids were initiated alongside antiviral therapy. Multimodal analgesia and antihistamines were also prescribed to provide symptomatic relief.

## Discussion

Primary varicella is now rare in adults due to widespread childhood vaccination, yet it remains an important diagnostic consideration, particularly in immunocompromised individuals. This case highlights the importance of maintaining a high index of suspicion for primary varicella in adults, regardless of vaccination history. 

Given the potential for severe disease and complications in adults, early recognition and appropriate management are imperative. Complications can include varicella pneumonia, hepatitis, encephalitis, disseminated varicella, and secondary bacterial infections [3]. While children are often not treated, adults are considered for antiviral treatment depending on the timing of presentation, and those with immunocompromise may require IV antiviral therapy. This is important as mortality rates can reach as high as 30% [10]. Given that our patient was clinically stable, the decision was made to initiate oral antiviral therapy with close follow-up and strict return precautions.

Patients with diabetes are more susceptible to complications like post-herpetic neuralgia and may have prolonged recovery periods. Diabetes mellitus is associated with immune dysfunction, including impaired T-cell-mediated immunity, neutrophil dysfunction, and chronic low-grade inflammation [11]. Since cell-mediated immunity is critical for controlling VZV, diabetic patients exhibit a higher risk of severe varicella, prolonged viremia, and complications such as secondary bacterial infections, pneumonia, and neurologic involvement [12]. This patient had diffuse cutaneous disease but no evidence of visceral involvement at the time of evaluation.

Like other viral exanthems, the rash of varicella is often preceded by a viral prodrome. However, the presence of vesicular lesions in different stages of healing, as seen in our patient, is a hallmark of varicella and can aid in distinguishing it from other vesicular rashes such as disseminated herpes simplex virus, smallpox, monkeypox, or other viral exanthems. Monkeypox lesions generally progress uniformly and are typically umbilicated with well-defined, regular borders; varicella lesions are usually more superficial with irregular edges [13]. Additionally, in monkeypox, fever typically begins one to three days before the rash and is accompanied by lymphadenopathy, whereas in primary varicella, fever usually starts one to two days before the rash, and lymphadenopathy is generally absent. 

Importantly, diagnosing varicella on skin of color presents unique challenges. Erythema may be more difficult to appreciate in darker skin tones, where it may present as a more violaceous hue. Additionally, skin of color is more prone to post-inflammatory hyperpigmentation, which can lead to longer-lasting visible skin changes following resolution of the rash.

## Conclusions

This case highlights the need for continued vigilance in recognizing primary varicella in adults, especially those with increased vulnerability due to underlying conditions. While the introduction of the varicella vaccine has led to a significant decline in adult varicella cases, patients with conditions that predispose them to impaired immune function remain at risk for severe disease and complications. Early recognition of atypical presentations and prompt initiation of antiviral therapy are critical to managing primary varicella and preventing serious adverse outcomes.
